# The Effect of Recombinant Erythropoietin on Plasma Brain Derived Neurotrophic Factor Levels in Patients with Affective Disorders: A Randomised Controlled Study

**DOI:** 10.1371/journal.pone.0127629

**Published:** 2015-05-26

**Authors:** Maj Vinberg, Kamilla Miskowiak, Pernille Hoejman, Maria Pedersen, Lars Vedel Kessing

**Affiliations:** 1 Psychiatric Centre Copenhagen, Rigshospitalet, University Hospital of Copenhagen Blegdamsvej 9, DK-2100 Copenhagen, Denmark; 2 Centre of Inflammation and Metabolism and Centre of Physical Activity Research, Rigshospitalet, 7641, Blegdamsvej 9, DK-2100 Copenhagen, Denmark; UNC Chapel Hill, UNITED STATES

## Abstract

The study aims to investigate the effect of repeated infusions of recombinant erythropoietin (EPO) on plasma brain derived neurotrophic factor (BDNF) levels in patients with affective disorders. In total, 83 patients were recruited: 40 currently depressed patients with treatment-resistant depression (TRD) (Hamilton Depression Rating Scale-17 items (HDRS-17) score >17) (study 1) and 43 patients with bipolar disorder (BD) in partial remission (HDRS-17 and Young Mania Rating Scale (YMRS) ≤ 14) (study 2). In both studies, patients were randomised to receive eight weekly EPO (Eprex; 40,000 IU) or saline (0.9% NaCl) infusions in a double-blind, placebo-controlled, parallel—group design. Plasma BDNF levels were measured at baseline and at weeks 5, 9 and at follow up, week 14. In contrast with our hypothesis, EPO down regulated plasma BDNF levels in patients with TRD (mean reduction at week 9 (95% CI): EPO 10.94 ng/l (4.51-21.41 ng/l); mean increase at week 9: Saline 0.52 ng/l, p=0.04 (-5.88-4.48 ng/l) p=0.04, partial ŋ^2^=0.12). No significant effects were found on BDNF levels in partially remitted patients with BD (p=0.35). The present effects of EPO on BDNF levels in patients with TRD point to a role of neurotrophic factors in the potential effects of EPO seen in TRD and BD. The neurobiological mechanisms underlying these effects and the interaction between EPO and peripheral levels on BDNF need to be further elucidated in human studies including a broad range of biomarkers.

**Trial Registration:**

ClinicalTrials.gov: NCT00916552.

## Introduction

Brain derived neurotrophic factor (BDNF) is a key target in the pathophysiology of neuropsychiatric disorders and the most widely distributed neurotrophin in the central nervous system where it plays several pivotal roles in synaptic plasticity, neuronal survival and immune system regulation [1]. Bipolar disorder (BD), recurrent depression and treatment resistant depression (TRD) are associated with dysregulation in the cellular signalling pathways that influence brain function and thereby behavioural performance but it is not fully elucidated whether peripheral BDNF potentially can serve as a biomarker in these disorders [2;3]. BDNF may be a critical modulator of neuroplasticity changes including neurogenesis. Hence, changes in BDNF may play a key role in the pathophysiology of mood disorders [4]. BDNF exerts potent effects on neuronal function and survival in various cell systems in the CNS, making it an intriguing candidate for development for neurological and psychiatric disease indications [5].

BDNF is present in the systemic circulation and can cross the blood-brain barrier, and at rest a cerebral output is seen in healthy young men [6]. It´s possible role has been widely studied in depression and to a lesser degree in other neuropsychiatric disorders. In brief, lower BDNF levels in serum and plasma have been reported in depression and BDNF levels seem to normalise levels in periods of remission [3]. In contrast, findings in BD are conflicting: some studies have demonstrated lowered levels of BDNF in manic or depressed bipolar patients compared with healthy control persons [7;8], while others have found increased BDNF levels [9;10]. Lower BDNF levels are also seen in schizophrenia [11] and in mild cognitive impairment and Alzheimer’s disorder [12]. BDNF may thus represent an unspecific biomarker of several different neuropsychiatric disorders with a neurodegenerative component. Interestingly, some chronic inflammatory disorders such as rheumatoid arthritis [13] and asthma [14] have been associated with elevated BDNF levels in the severe states. Finally, higher plasma BDNF levels seem to be associated with risk factors for cardiovascular disease including elevated diastolic blood pressure, higher cholesterol and higher BMI [15]. This suggests that the pattern of up- and down regulation of BDNF is complex and may be mediated through interrelations between different growth factors regulating the central nervous system.

The hematopoietic cytokine erythropoietin (EPO) acts as a cytoprotective agent in both neuronal and vascular systems which make it a candidate drug for neuroprotection [16]. Similar to BDNF, EPO is considered to have applicability in a variety of neuropsychiatric disorders [17] and it is capable of modulating multiple cellular signal transduction pathways to promote neuronal survival and enhance proliferation and differentiation of neuronal cells [18]. EPO crosses the blood-brain barrier [19] and is a potent growth factor that can protect CNS cells against apoptosis and promote proliferation of neuronal cells [18], thereby potentially being capable of preventing the progression of neurodegenerative processes in mood episodes. Evidence from preclinical studies, human neuroimaging studies, and recent clinical trials provide some indication for antidepressive cognitive enhancement effect, which might be mediated by action on EPO receptors located in the CNS [20]. In addition, we have demonstrated in two parallel clinical trials that EPO enhances cognition in patients with TRD and in patients with partial remitted BD [21;22].

In preclinical studies EPO seems to increase BDNF production [23;24] and BDNF gene expression in the brain [20;25] although studies are scarce. The effects of co-administered EPO on peripheral BDNF levels have never been studied in humans. In the present study, our primary hypothesis was that administration of EPO would be associated with increased BDNF levels in patients with TRD and in patients with BD in partial remission having cognitive difficulties.

## Methods

### Study design and participants

The study had a double-blind, placebo-controlled, parallel-group design which has been published elsewhere and described primary and secondary outcomes [21;22;26] (see summary pages 6–7). Patients were recruited through the Copenhagen Clinic for Affective Disorders, Psychiatric Centre Copenhagen, and by advertisement on relevant websites, and screened with Schedules for Clinical Assessment in Neuropsychiatry (SCAN) to confirm ICD-10 diagnosis [27]. Mood symptoms were assessed with the Hamilton Depression Rating Scale 17-items (HDRS-17) [28], Beck Depression Inventory 21-items (BDI-21) [29], and for bipolar patients also with the Young Mania Rating Scale (YMRS) [30] at baseline and weeks 5, 9, and 14. Cognitive function was investigated at baseline and weeks 9 and 14 with a comprehensive neuropsychological test battery including the Rey Auditory Verbal Learning Test (RAVLT).

Eligible patients had an ICD-10 diagnosis of TRD (defined as failure to respond to adequate treatment with at least two different types of antidepressants given in adequate time and doses according to the Treatment Response to Antidepressants Questionnaire (TRAQ) [31]) were moderately to severely depressed (HDRS-17 score ≥17) (study 1), or a diagnosis of BD in full or partial remission (HDRS-17 and YMRS scores ≤14) but with moderate to severe cognitive difficulties according to the Cognitive and Physical Functioning Questionnaire (CPFQ) [32] (score ≥4 on ≥2 domains) (study 2).

Exclusion criteria were significant medical conditions (diabetes, renal failure, epilepsy, hypertension, present or past malignancies, and thromboses), smoking, BMI >30, body weight <45 or >95 kg, schizophrenia, alcohol or substance misuse, acute suicidal risk, pregnancy or breast feeding, contraceptive medication, or a first-degree family history of thromboembolic events or seizure disorders. Benzodiazepines were tapered to a maximum of 22.5 mg oxazepam (or equivalent). Pregnancy tests were performed on female patients in their fertile age before and every second week during the study. Blood screening and physical examinations were undertaken at baseline and weekly during the treatment period, and at three follow-up visits to ensure patient safety. For an extensive description of the screening procedure, exclusion criteria, and safety precautions see [21, 22, 26]. Written informed consent was obtained from all patients before their inclusion and letters were sent to their general practitioners to rule out a history of significant medical conditions. The study was approved by The Capital Regions Local Ethics Committee: H-C-2008-092, Danish Medicines Agency (S2 Text) 2612–4020, EudraCT: 2008-04857-14, Danish Data Agency: 2008-41-2711 and ClinicalTrials.gov: NCT00916552.

### Randomisation and masking

Block randomisation was performed with stratification for age (< or ≥35 years) and gender. All outcome assessors were blinded to group assignment throughout the study, outcome assessment, data management, and analysis. The good clinical practice unit at Copenhagen University Hospital (www.gcp-enhed.dk/kbh) monitored that blinding was maintained and filed no report on any breach of blinding.

### Procedures

Patients were randomised to receive weekly infusions of EPO (Eprex; 40,000 IU; Janssen-Cilag) or saline (NaCl 0.9%) for eight weeks at the Clinic for Affective Disorders, Psychiatric Centre Copenhagen. Patients were randomized after the screening session and started the trial two weeks later for logistic reasons. They then received weekly intravenous infusions of either EPO (40 000 IU) or saline for eight weeks (weeks 1–8) in addition to their current antidepressant medication. This high dose and treatment schedule were chosen because several studies have shown that weekly administration of EPO in similar doses to other patient groups is effective for cognitive enhancement [33;34] and a single high dose to healthy volunteers enhances memory related dependent hippocampus response after one week [35]. Blood tests were taken at a weekly basis from baseline to week 10 (two weeks after treatment completion) and again in week 14. Mood symptoms were assessed at weeks 1, 5, 9, and 14 with the HDRS-17, BDI-21, and for bipolar patients also with the YMRS.

A summary of prior publications on the clinical primary outcome parameters is presented in [21,22]. In brief, the EPO-treated patients showed no reduction in depression severity as reflected by HDRS-17 scores (primary outcome) in comparison with saline in study 1 (TRD) [21]. Nevertheless, EPO-treated patients showed a reduction in self-rated depression on the Beck Depression Inventory (BDI) rating scale and improved quality of life. In addition, exploratory subgroup analyses of the patients who continued to fulfil the inclusion criteria at baseline (two weeks after the screening session) revealed significant improvement in HDRS-17 in the EPO over the saline groups [21]. In study 2 (BD), there was a trend towards improvement of verbal memory (primary outcome) in the EPO versus saline group, whereas EPO produced robust long-term enhancement of almost all additional measures of cognition including sustained attention, executive function, and overall speed of complex cognitive processing [22].

### Sampling and biochemical analyses

Blood samples were obtained at weeks 1, 5, 9, and 14 by venepuncture in the fasting state between 8.00 A.M. and 10.00 A.M before the clinical evaluation. To minimise BDNF-release from platelets, five millilitres of blood was drawn into a vacuum tube containing EDTA (Vacuette), which was kept on ice before and after blood drawing, and within 30 minutes centrifuged at 1590 g and 4°C for 15 minutes. Plasma was aliquoted into Eppendorf tubes and kept frozen at -80°C until assayed. Before analysis an additional centrifugation step of the separated plasma at 10,000 *g* for 10 minutes at 4°C was conducted.

Plasma concentration of BDNF was measured using a commercially available ELISA kit (QuantikineELISA, R&D Systems, Minneapolis, USA) according to the procedure provided by the manufacturer. Samples were analysed in duplicates and mean concentrations were calculated from a double-logaritmic fitted model standard curve. Investigators performing the analyses were blinded to treatment group. The detection limit for the BDNF assay was 58 pg/ml and few samples were below and excluded from analysis. Samples from the same participant were grouped together on the same plate in a randomly assigned sequence using the random numbers generator in SPSS, with an even distribution of samples from patients and healthy control subjects across plates. The intra-assay coefficient of variability (CV), as calculated based on results of the assays, was below 10%, while the inter-assay CV were 14.7%.

### Statistical analysis

The primary outcome in the present study was a change in BDNF levels from pre (baseline) to post treatment (week 9) as defined a priori in the trial presentation [21]. In further analyses BDNF levels at week 5 were added. Finally, the follow up visit six weeks after treatment completion was added to the analyses to observe whether there was a sustained effect on BDNF levels. Independent t-tests were used to test differences in clinical variables between the two groups, and the chi-squared test was used to examine differences in categorical demographic and clinical variables. Pearson’s correlations were used to analyse bivariate correlations. Comparative analyses between the groups were intention-to-treat (ITT) using last observation carried forward (LOCF) for missing values. Data was analysed with repeated measures analysis of covariance (ANCOVA) with adjustment for stratification variables to minimise effects of any baseline imbalances. A logarithmic transformation was performed on BDNF data because of the more stable standard deviations. Transformed data were used in all subsequent analyses. Differences were reported differences in means, 95% CIs, p-values, and effect size as reflected by partial partial ŋ^2^. The Statistical Package for the Social Sciences was used for the statistical analyses (SPSS, version 19 for IBM).

## Results

### Study characteristics

**Fig 1:**
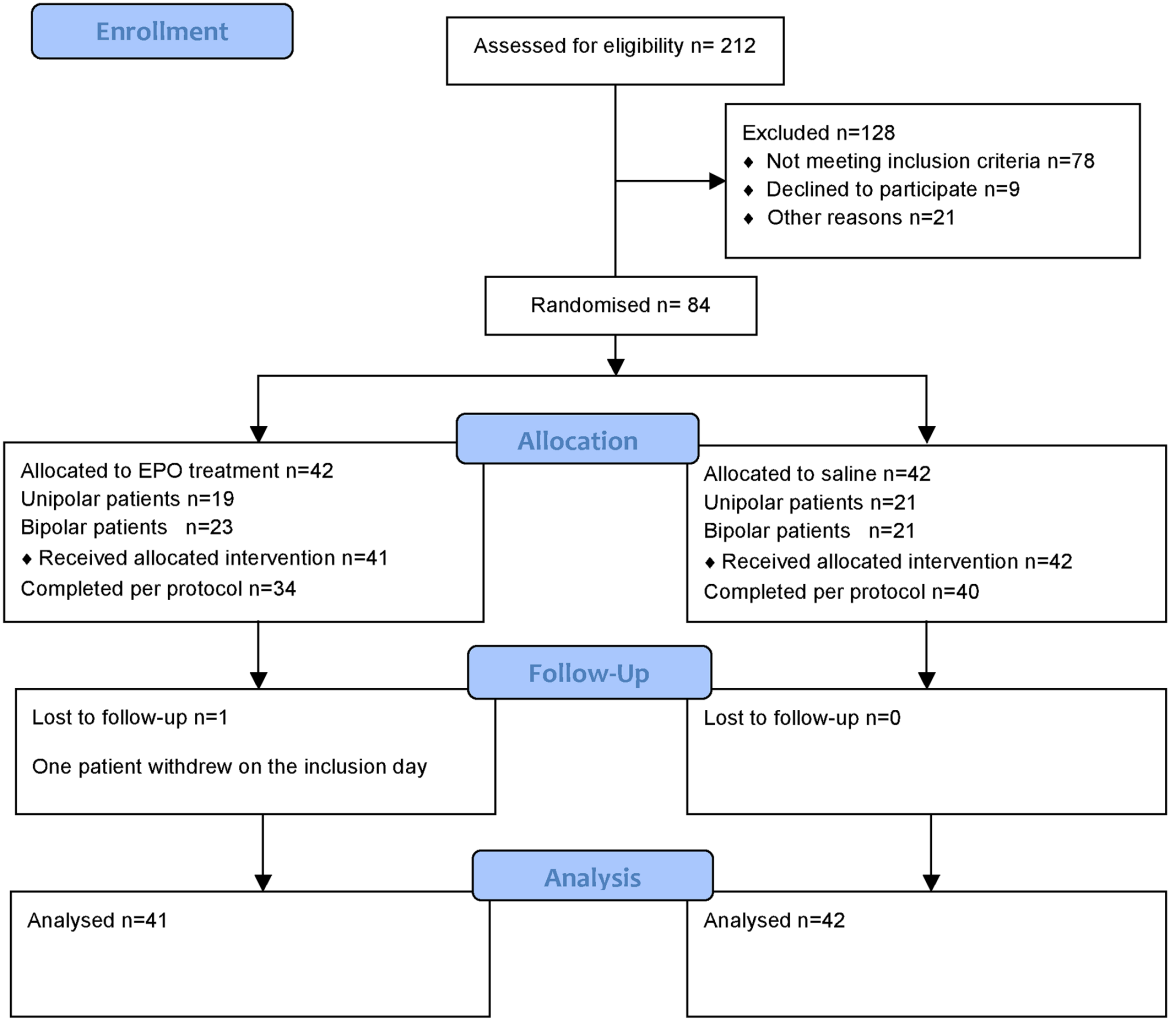
CONSORT Flow Diagram.

Patients for both studies were included and randomised from September 2009 to October 2012. In total, 212 patients were assessed and 128 were excluded (Fig 1, S1 Text) Of the 84 randomised patients, one patient withdrew on the inclusion day, leaving 83 patients for analyses (EPO, N = 41; saline N = 42), of whom 74 completed per protocol (PP) (EPO, N = 34, saline N = 40). Of the nine patients who did not complete PP, six patients (EPO) discontinued medication after 5–6 infusions because of increased platelet count (> 4 x 10^9^/l) but completed all assessments and three patients (one EPO week 6, one EPO week 10, one placebo, week 10) were admitted to the hospital because of acute suicide risk. LOCF was thus performed from week 5 (saline) on one patient and from weeks 9 to 14 for two patients (EPO).

As can be seen from Table 1 and Table 2, the two groups were well matched in terms of age, gender, and baseline characteristics. All patients continued their medication as usual and all but four received medication for their diagnoses and were not allowed to change their medication in the study period.

**Table 1:** Patients with unipolar treatment resistant depression characteristics.

	Epo group N = 18	Saline group N = 21
Age, Years	41 (9)	45 (14)
Gender, no. female (%)	13 (72)	14 (66)
Years of education	15 (3)	15 (3)
BMI	25 (3)	26 (3)
HDRS-17 score at baseline	20 (4)	20 (4)
HDRS-17 score week 9	15 (6)	16 (6)
RAVLT total score baseline	44 (11)	43 (9)
RAVLT total score week 9Number of previous Depressiove Episodes	50 (9)	45 (11)
Number of adequate treatmens with different classes of antidepressants according to the TRAQ*	4 (1)	4 (1)
Patients previously treated with ECT, No. (%)	3 (17)	5 (24)
Medication during the trial period **		
Number of medications	2.3 (1.7)	2.5 (1.4)
SSRI	5	6
Dual Action SNRI	3	9
Antipsychotics	5	6
MAOI	1	0
TCA	2	1
Sleeping medication	2	1
Benzodiazepines	1	5
Lithium	1	3
No medication	2	1

In brackets: Mean Standard Deviation

Abbreviations: EPO: erythropoietin; BMI: body mass index; HDRS-17: Hamilton Depression Rating Scale 17 items; TRAQ: Treatment Response to Antidepressants Questionnaire; ECT: electroconvulsive treatment, SSRI: Selective Serotonin Reuptake Inhibitors, SNRI: Selective Noradrenaline Reuptake Inhibitors, MAOI: Monoamine Oxidase Inhibitors, TCA: Tricyclic Antidepressants.

* Medical treatment history was evaluated using treatment response to antidepressants questionnaire (TRAQ) (Posternak, 2004). Each antidepressant medication and each combination of different antidepressants or add-on treatment with other classes of drugs were assessed as separate antidepressants trials.

**No patients made changes in their medication from weeks 1–9; after week 9, medication change was performed for eight patients (EPO: N = 2; saline: N = 6).

**Table 2:** Patients with bipolar disorder characteristics.

	Epo group N = 23	Saline group N = 21
Age, Years	41 (13)	40 (11)
Gender, no. female (%)	14 (61)	13 (62)
Years of education	15 (4)	14 (4)
BMI	24 (2)	25(3)
HDRS-17 score at baseline	10 (4)	9 (2)
YMRS score at baseline	3 (2)	3 (2)
RAVLT total score baseline	46 (9)	50 (9)
RAVLT total score week 9	49 (10)	50 (11)
Bipolar I diagnosis, no. (%)	10 (44)	8 (38)
Number of previous Depressiove Episodes	7 (7)	5 (4)
Number of Previous Manic Episodes	1 (2)	1 (2)
Number of Previous Hypomanic Episodes	6 (8)	3 (4)
Medication during the trial period		
Lithium, no. (%)	12 (52)	5 (24)
Anticonvulsants, no. (%)	13 (57)	11 (52)
Antidepressants, no. (%)	10 (43)	11 (52)
Antipsychotics, no. (%)	6 (26)	4 (19)
Benzodiazepines, no. (%)	10 (43)	7 (33)
Melatonin, no. (%)	1	1
No medication, no. (%)	0 (0)	1 (5)
Number of medications	2.4 (1.0)	2.0 (0.8)

In brackets: Mean Standard Deviation

Abbreviations: EPO: erythropoietin; BMI: body mass index; HDRS-17: Hamilton Depression Rating Scale 17 items; YMRS Young Mania Rating Scale.

### BDNF levels in patients with TRD

**Fig 2:**
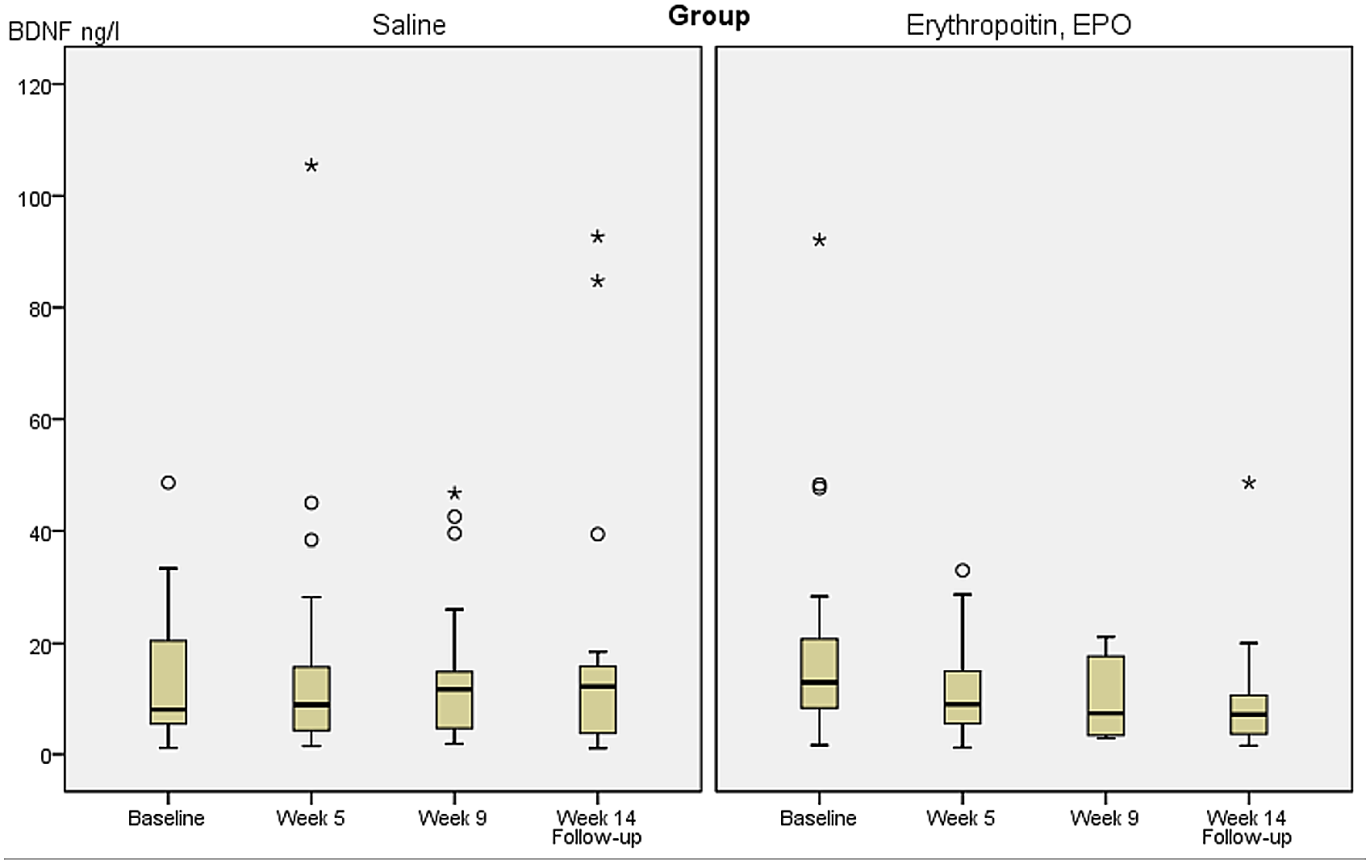
Plasma Brain Derived Neurotrophic Factor (BDNF) levels according to treatment group in patients with treatment resistant depression.

BDNF levels at baseline, and weeks 5, 9, and at follow-up week 14 are presented in Table 3 and shown according to treatment group in Fig 2. Independent t-test comparing BDNF levels at revealed no significant differences between the two groups at any time point (p ≥ 0.21). In further analyses, using within subject dependent t-tests to compare baseline with endpoint (week 9) measures within each group separately revealed a significant reduction in BDNF levels over time in the EPO group (baseline BDNF levels: mean 20.87 ng/l, SD 22.02 versus BDNF levels at week 9: 9.94 ng/l, SD 7.04, p = 0.04, df = 18). In contrast, the Saline group showed no change in BDNF levels from baseline to week 9 (baseline BDNF level: 13.75 ng/l, SD 12.43 versus BDNF levels at week 9: 14.28 SD 13.52, p = 0.84, df = 21).

**Table 3:** Results for all unipolar patients with treatment resistant depression (N = 39), df (1,39). BDNF levels ng/l (in brackets, Mean Standard Deviation).

	Week 1 baseline	Week 5	Week 9	Week 14	Time (weeks 1,9) P* Time (weeks 1,9) by treatment group P-values	Time (weeks 1-5-9) P* Time (weeks 1, 5, 9) by treatment group P-values
EPO (N = 18)	20.88 (22.02)	8.67 (2.04)	9.94 (7.04)	10.23 (10.95)	P = 0.36* F = 0.36	P = 0.21* F = 0.62
Saline (N = 21)	13.76 (12.43)	16.43 23.56	14.28 (13.53)	18.41 (24.87)	P = 0.04 F = 4.57	P = 0.059 F = 3.07

At baseline (week 1), half-way through treatment (week 5), and upon treatment completion (week 9) and follow-up week 14. Factor time, P* and factor time by treatment group interaction including baseline and week 9 BDNF levels (weeks 1,9) and baseline, week 5, and week 9 BDNF levels (weeks 1-5-9). Covariates for repeated-measures ANCOVA in all analyses: age and gender.

As seen from Table 3, in repeated measures analysis of covariance (ANCOVA), BDNF levels decreased significantly in the EPO versus saline treated patients from weeks 1 to 9 (mean reduction (95% CI): EPO group 10.94 (4.51–21.41 ng/l), mean increase: Saline group 0.52 (-5.88–4.48 ng/l), df(1,39) F = 4.57, p = 0.04, partial ŋ^2^ = 0.12). Using Bonferroni correction to account for chance findings in multiple comparisons, the p-value was reduced to a trend level (p = 0.07, partial ŋ^2^ = 0.09). In further analyses, repeating the ANCOVA analyses including BDNF levels at week 5 the association was reduced to a strong trend (F = 3.07, p = 0.059, partial ŋ^2^ = 0.08). Finally, in order to test whether the effect remained after discontinuation of the trial medication, adding the follow-up visit in week 14, six weeks after treatment completion, there was still a trend towards reduction in BDNF levels in the EPO-treated TRD patients (F = 2.33, p = 0.098, partial ŋ^2^ = 0.06). In contrast, repeated measures ANCOVA revealed no general changes in BDNF levels over time (Table 3*).

### BDNF levels in patients with BD in partial remission

BDNF levels at baseline, weeks 5, 9, and at follow-up week 14 are presented in Table 4. Independent t-test comparing BDNF levels at baseline revealed no significant differences between groups (p = 0.83). Using repeated measures ANCOVA in the group of patients with BD there was no differential change in BDNF levels between the two treatment groups over time (p≥0.35). Repeated measures ANCOVA revealed no general changes in BDNF levels over time (Table 4*)

**Table 4:** Results for all patients with bipolar disorder (N = 44), df (1, 44). BDNF levels ng/l (in brackets, Mean Standard Deviation).

	Week 1 baseline	Week 5	Week 9	Week 14	Time (weeks 1,9) P* Time (weeks 1,9) by treatment group P-values	Time (weeks 1-5-9) P* Time (weeks 1, 5, 9) by treatment group P-values
EPO (N = 23)	20.42 (30.07)	10.03 (13.38)	13.74 (23.81)	9.63 (9.71)	P = 0.45* F = 0.36	P = 0.74* F = 0.31
Saline (N = 21)	18.71 (19.93)	10.49 (9.66)	10.36 (12.30)	7.97 (6.13)	P = 0.35 F = 0.90	P = 0.50 F = 0.71

At baseline (week 1), half-way through treatment (week 5), and upon treatment completion (week 9) and follow-up week 14,. Factor time, P* and factor time by treatment group interaction including baseline and week 9 BDNF levels (weeks 1,9) and baseline, week 5, and week 9 BDNF levels (weeks 1-5-9). Covariates for repeated-measures ANCOVA in all analyses: age and gender.

### Exploratory analyses

Conducting post hoc exploratory analyses adjusted for parameters known to affect difference in BDNF (differences in depression severity (HDRS-17 scores) and verbal memory (total RAVLT scores) between baseline and at week 9,) did not reveal any significant associations in either the patients with TRD or in the patients with BD. Pearsons correlation analyses between BDNF levels and HDRS-17 and total RAVLT scores at baseline, weeks 5, 9, and 14, respectively, did not reveal any significant correlations (results not presented). Adjusting for prior antidepressant treatments in post hoc analysis revealed that the number of adequate prior treatments with different classes of antidepressants contributed significantly to lower BDNF levels at week 9 in patients with TRD (F = 7.63, p = 0.01,). In further post hoc analyses concerning study 1, excluding all data point with BDNF levels ≥ 30 ng/l, the significant difference was reduced to a strong trend (F = 3.70, p = 0.063). Although there were no significant differences between the two TRD groups at baseline, a post analyses adding baseline BDNF levels as a covariate was conducted. Baseline BDNF levels did contribute significantly to lower BDNF levels at week 9 (F = 11.19, p = 0.02) reducing the difference between the two groups to a trend level (F = 3.17, p = 0.084).

As EPO increases thrombocyte levels, a post hoc analysis adding the variable: difference in platelet count between baseline and week 9 was conducted. This difference contributed significantly to the decreased BDNF levels seen in patients with TRD (platelets baseline, Saline: 264, SD 58, EPO: 259, SD 74, platelets week 9, EPO: 294, SD 59, Saline: 265, SD 60, F = 11.25, p = 0.01), but the effect of treatment group remained significant (F = 12.19, p = 0.001). In patients with BD no significant effect was shown when adding the difference in platelet count (platelet count baseline, Saline: 256, SD 68, EPO: 257, SD 56, platelet count week 9, Saline: 243, SD 60, EPO: 292, SD 67). Finally, as EPO also increased haematocrit levels [36], a post hoc ANCOVA analyses, adding the difference in hematocrit levels between baseline and week 9, as a covariate, was conducted. This revealed no significant associations between differences in hematocrit levels and BDNF levels either in the patients with TRD or the patients with BD (results not shown).

## Discussion

The present study explored the effect of EPO on peripheral levels of BDNF in patients with affective disorders. In contrast with our hypothesis EPO reduced plasma BDNF levels in patients with TRD while no effects were observed in patients with partially remitted BD. These associations were not influenced by age, gender, baseline depression severity, change in depression severity, or memory performance from baseline to week 9. Further, lower BDNF levels were associated with the number of prior antidepressant treatment trials in patients with TRD, platelet count and baseline BDNF levels.

Interestingly, lower BDNF levels were associated with the number of prior antidepressant treatment trials in the patients with TRD. Unfortunately, it was not possible to conduct this analysis in the bipolar patient cohort as we did not assess prior medical treatments trials for these patients. Medication may alter neurotrophic signalling; a number of studies have investigated the effect of antidepressants on peripheral BDNF levels in patients with depression and showed that the use of SSRIs is associated with an increase in peripheral BDNF levels [37;38]. The same pattern was observed using lithium in unipolar depression [39]. However, BD studies are scarce and the results conflicting. One study showed that quetiapine used in acute mood episodes increased BDNF levels in patients with a depressive episode but decreased BDNF levels in patients with a manic/mixed episode [40]. Another study showed that four weeks of treatment with risperidone had no effect on BDNF levels [41]. Eleven patients in the EPO group and 12 patients in the saline group were prescribed benzodiazepines. However, only a few patients received benzodiazepines on a daily basis, and most used benzodiazepines as necessary to a maximum of 22.5 mg oxazepam (or equivalent and they were encouraged to avoid using benzodiazepines 24 hours before assessment). In the present study, almost all patients were in medical treatment, and the different treatment groups were equally distributed across the two groups (Table 1), except in the TRD group (study 1), where nine saline-treated patients and three EPO-treated patients were treated with dual action antidepressants, minimising the possibility that the present findings could be due to differences in the medication. In contrast with the present study, a metaanalysis on the effect of electroconvulsive therapy (ECT) compromising result from 11 studies (n = 221 patients) with unipolar disorder, BD or psychotic depression showed that peripheral BNDF increased after ECT treatment [42].

No previous studies have investigated the effect of EPO on BDNF levels in humans. We hypothesised that EPO would increase peripheral BDNF levels since EPO infusion, as previously mentioned, increases CNS BDNF in preclinical models, and in several human studies of patients with neurodegenerative and neuropsychiatric conditions EPO is also associated with neuroprotective and neuroproliferative potentials [16], including TRD and BD [21;22]. Indeed, we demonstrated with prospective magnetic resonance imaging (MRI) that EPO also prevents brain matter loss in a subfield of the left hippocampus and that this volume increase was associated with improvement of memory function in the present cohort of patients with TRD and BD [36]. Whether this is due to a general cellular proliferation or possibly angiogenesis is unresolved although increased hematocrit levels in the EPO group had normalised at the time of the second MRI scan (performed at the follow-up visit in week 14). The increased hemotocrit level during the active study period had no effect on BDNF levels in the present study. Nevertheless, one may speculate whether EPO may mediate its effect directly on the mammalian target of rapamycin (mTOR) pathway which is a crucial regulator of cell growth and proliferation. Further, a mTOR activation stimulates both cell growth, angiogenesis, and cell migration as illustrated in preclinical studies showing that EPO seems to mediate it´s effects via the mTOR signalling pathway [43;44].

Thus, the beneficial effects of EPO reported in our primary analyses [21;22] may not be driven through the effect of EPO on BDNF-driven signal transduction pathways. Conversely, it may be plausible that increased BDNF levels are not uniformly associated with a beneficial outcome and neuroprotective effects. Indeed, a study from our group demonstrated that bipolar patients with severe course of rapid cycling had significant increased BDNF levels as compared with age and gender matched control persons [45] which was in line with another recent study also including bipolar patients with a severe course [9]. Further, as described in the introduction elevated BDNF levels seem related to chronic inflammation e.g. rheumatoid arthritis and asthma and are also associated with increased risk factors of cardiovascular diseases. In the present study the decreased BDNF levels were only seen in currently depressed patients with TRD. This finding may relate to the symptom level, and thus the decrease may correlate with the differences in HDRS-17 scores. Nevertheless, the decreased BDNF levels in the TRD cohort were not associated with change in HDRS-17 scores from visits 1 to 9, and we found no correlation between HDRS-17 scores and BDNF levels on any of the four visits. This is in line with previous studies from our group [46] and others [47]. In contrast, a study of inpatients showed that BDNF plasma levels were significantly lower in the patients with the most severe illness [48]. In the present study BDNF levels were also not correlated with changes in memory performance score. In line with a study also including patients with major depressive disorder, this study showed no correlation between cognitive performances and BDNF levels [49]. Additionally, a longitudinal study showed no association between serum BDNF and subsequent cognitive test trajectories, in older adults, although a potential trend towards a cross-sectional association was found [50].

Within the total cohort, BDNF levels tended to decrease over time especially between the baseline visit and week 5 visit, and this decrease was attenuated in the EPO-treated group. However, the influence of time was not significant, whereas another longer follow-up study found a significant decrease in BDNF over time [51]. Concerning study 2, including patients with BD in partial remission, it cannot be excluded that the effect of EPO versus placebo on BDNF levels would be different among patients in a severe depressive state in line with a recent review pointing at peripheral BDNF levels as a biomarker of disease activity [52].

The cellular source of BDNF would influence the interpretation of the present finding as it is still unresolved whether peripheral changes in BDNF levels reflect central BDNF regulated neuroplasticity. In circulation, BDNF is primarily present in platelet granules and is released upon platelet activation [53]. Consequently, one explanation for the present finding could be due to the increased platelet levels seen in the EPO-treated group as the decreased BDNF levels may be a consequence of the increase in platelet count. Thus, the EPO-induced platelets would assimilate circulating BDNF. Indeed, post hoc analyses adding the variable: change in platelet count contributed significantly to the decreased BDNF levels seen in patients with TRD, but the effect of the treatment group remained significant, suggesting that the increased platelet count was not the whole explanation. EPO may then directly reduce the BDNF release from platelets in line with preclinical studies showing that BDNF release from platelets is affected by antidepressants [54;55]. Further, EPO is a cytokine and capable of preventing inflammation [17]. Hence, the present result of a significant decreased BDNF levels in currently depressed patients with TRD, could also be mediated through EPO´s effects on the inflammatory pathway. Given the present result it would be of interest to investigate possible correlations between inflammatory markers (e.g. IL-6, Il-10 and high sensitive C-reactive protein) and BDNF levels in the present cohort.

### Strengths and limitations

A strength of the present study is the use of fasting samples collected at the same time of day and based on rather strict inclusions criteria according to the EPO´s haematopoietic side-effects, not influenced by smoking, alcohol abuse, high BMI, use of contraceptive pills, or concurrent somatic disorders. It is an additional strength that nearly all participants fulfilled per protocol although six participants did not receive all eight EPO injections due increased risk of thrombosis, reflected by elevated platelet levels to above normal range (removing these six individuals in post hoc analyses did not change the results). The relatively few participants (n = 83) is a limitation, and BDNF levels also exhibit a large intra- individual heterogeneity, and the significant difference in the TRD study was reduced to a strong trend when removing outliers. Finally, the significant finding was, when corrected for multiplicity, reduced to a trend level. The present finding should therefore be considered as exploratory and be interpreted with caution. Concerning the present finding of a reduced BDNF level in the EPO group in patients with TRD, a post hoc statistical power calculations using the open source statistical power calculation tool https://www.dssresearch.com/KnowledgeCenter/toolkitcalculators/statisticalpowercalculators.aspx showed our sample size of n = 39 with mean (SD) BDNF levels of 17.3 ng/l (17.2) provided a power of 0.93 to detect a significant difference between the groups at a significant level of 0.05 (two-tailed), if we assume a mean difference in BDNF levels of 7.0 ng/l between the two groups, discriminates between those who received EPO and those who received saline. If we assume that 10.0 ng/l is a clinical relevant difference the power was reduced to 0.76. Further, we did not register daily physical exercise level, and since EPO is well known for its potential doping capacity the change in BDNF levels could be due to increased exercise levels in the intervention group. Nevertheless, there are conflicting results on how physical activity influences BDNF levels. A review showed a positive effect of exercise on BDNF levels in healthy participants [56], while a small study on depressed patients showed no changes in BDNF levels [57] One study has shown that long-term exercise may in fact lower BDNF levels in healthy participants [58].

Currently, the impact of prior course of illness is not clarified; one study revealed a difference in peripheral BDNF levels between patients with a first-episode depression and patients with recurrent depression [59], whereas two other studies did not find an effect of prior course of illness [60;61]. In the present study, post-hoc analyses adding the number of previous episodes as an independent variable did not influence the results. Nevertheless, as both patient cohorts were comprised mainly of patients with a more severe and longer course, the influence of EPO on BDNF levels may differ in patients in the early versus late/chronic course of the disorders. Further, the evidence that episodes of depression are associated with low peripheral BDNF is mostly based on cross sectional data and studies following patients over time are mostly follow-up studies including an active medical treatment (for review [3;7]). Our finding that the number of previous antidepressant treatments contributed to decreased BDNF levels at follow-up in patients with TRD possibly indicates that peripheral BDNF levels may interact with treatment duration. BDNF levels may thus act differently in early stages compared with late stages of the disorders, an assumption also stated in a review on circulating levels of BDNF in neuropsychiatric disorders [12].

## Conclusions

EPO down regulated plasma BDNF levels in patients with TRD, whereas no effect was observed in patients with BD. Future human studies, should include more patients cross diagnoses and healthy control persons: It is further recommended to integrate a broad range of biomarkers to disentangle the complex interrelations between the different growth factors regulating the central nervous system.

## Supporting Information

S1 Text CONSORT 2010 Checklist. (PDF)

S2 Text Study Protocol. (PDF)
