# Supplementary material for: The Effect of Recombinant Erythropoietin on Plasma Brain Derived Neurotrophic Factor Levels in Patients with Affective Disorders: A Randomised Controlled Study
Source: PLoS One. 2015 May 26;10(5):e0127629. doi: 10.1371/journal.pone.0127629 (PMC4444304; doi:10.1371/journal.pone.0127629)
Supplement: S2 Text — (PDF) [file pone.0127629.s002.pdf]

Effekt af erythropoietin på depressive symptomer og neurokognitive deficit ved behandlingsresistent depression og ved bipolar lidelse i remitteret fase – et proof of concept studie.

Dr Kamilla W. Miskowiak[1,2], Dr Maj Vinberg[1], Dr Catherine J. Harmer[2], Prof Hannelore Ehrenreich[3], Prof Gitte M. Knudsen[4], Prof Olaf B. Paulson[4,5] Prof Lars V. Kessing[1]

[1]Kompetencecenter for Affektivel Lidelser, Region Hovedstadens Psykiatri, Psykiatrisk Center Rigshospitalet, København
[2]Department of Psychiatry, University of Oxford, Warneford Hospital, Oxford
[3]Max-Plancks Institute Institute of Experimental Medicine, Göttingen
[4]Neurobiologisk Forskningsenhed (NRU), Rigshospitalet, København
[5]MR-Afdelingen, Region Hovedstaden Hvidovre Hospital, Hvidovre

Resumé

Depression og bipolar lidelse er associeret med neurodegenerative processer, reduceret neural plasticitet og neuropsykologiske dysfunktioner, som ofte vedvarer selv efter klinisk remission. Nuværende farmakologiske behandlingsstrategier har adskillige begrænsninger, herunder betydelig latenstid indtil klinisk virkning, til tider manglende effekt og begrænset virkning på kognitive deficit. Erythropoietin (Epo) har neurotrofiske og neuroprotektive egenskaber i dyremodeller. I overensstemmelse med dette har vi påvist, at en enkelt intravenøs dosis af Epo til mennesker har en positiv virkning på neurokognitive funktioner og stemningsleje (Miskowiak et al 2007a, 2007b, 2008a, 2008b). Vi ønsker med det aktuelle proof-of-concept studie at udbygge disse fund ved at undersøge, om ugentlig Epo administration over 8 uger har antidepressiv virkning hos patienter med behandlingsresistent depression og er i stand til at normalisere vedvarende neurokognitiv dysfunktion hos disse patienter og hos patienter med bipolar lidelse i remitteret fase. Hvis vores forskning viser gavnlig virkning af Epo på neurokognitive funktioner og stemningslejet i disse patientgrupper, vil det tale for klinisk relevans af Epo som tillægsbehandling ved affektive lidelser.

Baggrund

Behandling af affektive lidelser og neuroplasticitet
Depression og bipolar lidelse (depression og mani) er ifølge Verdens Sundheds Organisationen (WHO) den hhv. største og femte største årsag til den globale sygdomsbelastning i form af sygdomsvarighed (antal år levet med sygdom) (WHO 2001). Disse affektive lidelser har svære implikationer for livskvalitet og udgør en stor økonomisk byrde med arbejdsløshed og udgifter for sundhedsvæsenet. Der er derfor et presserende behov for udvikling af bedre og mere effektive behandlingstilbud til disse patientgrupper.

De nuværende behandlingsstrategier har stadig mangler. Konventionel antidepressiv behandling er flere uger om at virke og er kun delvist eller slet ikke effektiv for en stor patientgruppe. Derudover virker eksisterende medikamentel behandling kun delvist på neurokognitiv deficit ved depression og bipolar lidelse (for oversigtsartikler se Marvel & Paradiso 2004; Martínez-Arán et al 2000; Clark & Goodwin 2004; Chamberlain & Sahakian 2004, 2006), som varer ved selv efter klinisk remission og menes at være en central årsag til patienternes forringede sociale og arbejdsmæssige funktionsniveau (Dickerson et al 2001; Dion et al 1988). Bedre behandlingsmuligheder er derfor påkrævet for at reducere tiden til den kliniske effekt, for at hjælpe de hidtil behandlingsresistente patienter og for at forbedre de neurokognitive funktioner hos patienter med bipolar lidelse i remitteret fase.

Meget tyder på, at neural atrofi og reduceret neuroplasticitet er involveret i udviklingen af unipolar og bipolar lidelse, mens øget neural plasticitet er en nøglemekanisme i vellykket antidepressiv og stemningsstabiliserende behandling (for oversigsartikler, se Berton & Nestler 2006; Belmaker & Agam 2008; Manji & Duman 2001; Frey et al 2007). Denne "neurotrofiske hypotese" er støttet af, at øgning i brain-derived-neurotrophic-factor (BDNF) og neurogenese i hippocampus er nødvendigt for adfærdsmæssige virkninger af antidepressiv behandling (Santarelli et al 2003), og at infusion af BDNF i hippocampus imiterer de adfærdsmæssige effekter af antidepressiva i gnavere (Shirayama et al 2002). Disse virkninger og rollen af BDNF i indlæring og hukommelse tyder på, at præperater, som inducerer hurtig og vedvarende opregulering af BDNF og neuroplasticitet kan reducere latenstiden til antidepressiv virkning og genoprette neurokognitive funktioner.

Epo og neuroplasticitet
Epo er bedst kendt for sin rolle i dannelsen af røde blodlegemer, men bliver udover i nyrerne også produceret i hjernen og udøver der vigtige neurobiologiske virkninger. Parenteral administreret Epo trænger ind gennem blod-hjerne-barrieren og har neuroprotektive og neurotrofiske virkninger i dyremodeller for akut hjerneskade og kroniske neurodegenerative lidelser (Morishita et al 1997; Sakanaka et al 1998; Brines et al 2000; Sättler et al 2004; Yatsiv et al 2006; Lee et al 2006; Agnello et al 2002). Adskillige mekanismer medierer de neuroprotektive virkninger inklusiv opregulering af BDNF og neurogenese (Wang et al 2004; Viviani et al 2005; Ransome & Turnley 2007) samt reduktion af neural apoptose og inflammation (Yatsiv et al 2006; Lee et al 2006; Agnello et al 2002; Sättler et al 2004). Studier af Epos virkning på hjernefunktion hos mennesker har vist, at Epo behandling påbegyndt inden for 8 timer efter iskæmisk hjerneinfarkt reducerer infarkt størrelse og forbedrer neurologisk genopretning (Ehrenreich et al 2002). Endvidere forbedrer langtids Epo-behandling kognitive funktioner hos patienter med skizofreni (Ehrenreich et al 2007a) og multipel sklerose (Ehrenreich et al 2007b), hvilket tyder på en rolle af Epo som "kognitiv enhancer" i forskellige psykiatriske og neurodegenerative forstyrrelser. På grund af samtidig øgning af antallet af røde blodlegemer er det imidlertid usikkert, om Epos virkning er direkte eller indirekte ved øget ilttilførsel til hjernen.

Vi har derfor foretaget funktionel MR skanning og kognitiv testning af raske mennesker efter en enkelt dosis af Epo versus placebo i fravær af hæmatologiske effekter (Miskowiak et al 2007a, 2007b, 2008a, 2008b). En uge efter dets administration øgede Epo den neurale aktivering i hippocampus under billedgenkendelse (Miskowiak et al 2007a), hvilket kan forklares med øget hippokampal neuroplasticitet, BDNF og neurogenese (Hariri et al 2003; Ransome & Turnley 2007). Epo påvirkede endvidere den neurale mediering af arbejdshukommelse og forbedrede ordmobilisering (Miskowiak et al 2008b). Udover disse positive neurokognitive virkninger, nedregulerede Epo neural og psykologisk respons til negative emotionelle informationer (Miskowiak et al 2007b) på samme måde, som serotonerge og noradrenerge antidepressiva (Harmer et al 2004, 2006) og forbedrede stemningslejet i de første tre dage efter dets administration. Preliminære resultater fra vores pågående undersøgelse af 19 depressive patienter bekræfter disse antidepressiv-lignende virkninger af Epo på bearbejdning af emotionelle informationer tre dage efter dets administration (Miskowiak et al, under udarbejdelse). Epo synes således at kunne direkte modulere emotionel processering i akut syge patienter på en måde, der ligner virkningen af konventionel antidepressiv behandling. Ud fra disse resultater er det oplagt at undersøge Epos virkning på neurokognitive funktioner og stemningsleje i patienter med depression og, om Epo på sigt kunne blive anvendt som 'kognitiv enhancement' tillægsbehandling ved sværere depressive tilstande samt ved remitteret unipolar og bipolar lidelse karakteriseret ved vedvarende neurokognitive deficit.

Mulige biomarkører for behandlingseffekt
I overensstemmelse med den neurotrofiske hypotese er nedsat perifer BDNF påvist hos ubehandlede patienter med unipolar og bipolar lidelse (Shimizu et al 2003; Karege et al 2002, 2005; Gonul et al 2005; Cunha et al 2006), unipolare patienter i antidepressiv behandling

(Grassi-Oliveira et al 2008) samt hos euthyme patienter med affektive lidelser uafhængigt af medicinsk behandlingsstatus (Monteleone et al 2008). Endvidere er der fundet en negativ korrelation mellem serum BDNF og depressive symptomer målt med Hamilton Depression Scale (HAMD; Hamilton 1960) hos depressive patienter (Yukimasa et al 2006; Yoshimura et al 2007). Det er derfor blevet postuleret, at øget serum/ plasma BDNF kan være en biologisk markør for klinisk remission fra depressive tilstande (Mitoma et al 2008). Denne hypotese understøttes af evidens for nedsat perifer BDNF hos patienter med bipolar lidelse (Shimizu et al 2003; Karege et al 2002, 2005; Grassi-Oliveira et al 2008; Gonul et al 2005; Cunha et al 2006) og korrelerer med depressive symptomer (Yukimasa et al 2006; Yoshimura et al 2007). Prækliniske studier har vist, at Epo øger BDNF i CNS (Viviani et al 2005; Wang et al 2004), men effekten af Epo-behandling på perifær BDNF hos patienter med depression er ukendt.

En anden markør for klinisk remission er den kognitive processering af emotionelle informationer. Depression ledsages af negative bias i opmærksomhed og hukommelse for emotionelle informationer, og disse bias korrelerer positivt med depressionens sværhedsgrad og varighed (Bradley et al 1995). Ny forskning viser, at antidepressiva producerer tidlige forandringer i den relative processering af positive vs. negative informationer, som går forud for kliniske ændringer i stemningslejet (Harmer 2008). Mere specifikt, reducerer antidepressiva den kognitive og neurale processering af negative emotionelle informationer, hvilket menes at danne grundlag for efterfølgende kognitiv omstrukturering og dermed bidrage til bedring af stemningslejet. Vores adfærdsmæssige og billeddannelses-data peger på, at Epo nedregulerer processeringen af angste ansigtsudtryk hos raske (Miskowiak et al 2007b) og depressive individer (Miskowiak et al, submitted), på samme måde som nuværende antidepressiva (Harmer 2008). Det er dog uvist, om sådanne ændringer korrelerer med klinisk ændring af stemningslejet hos patienter med affektive lidelser.

I de seneste år har der været en stigende interesse i sammenhængen mellem affektive lidelser og metabolisme. Metabolisk syndrom er påvist hos 25-50% af patienter med bipolar lidelse og hos 12-36% af patienter med tilbagevendende depression (for oversigtsartikel, se Jakovjevic et al 2007). Ifølge en ny hypotese spiller ændringer i metaboliske funktioner en fundamental rolle i fysiopatologien ved affektive lidelser (McIntyre et al 2007), skønt mekanismerne endnu er ukendte. Det er derfor blevet foreslået, at medicin med indvirkning på metaboliske processer kan udgøre en ny generation af antidepressiva (ibid.). I forsøg med mus, hvor EPO blev over-udtrykt i høje koncentrationer, er der fundet en markant reduktion (28%) i fedtmassen hos både slanke mus og mus på en fedtholdig diæt (Hojman et al, submitted). Denne ændring i fedtmasse blev ledsaget af en fuldstændig normalisering af forskellige metaboliske parametre herunder insulinsensitiviteten. Et enkelt studie har undersøgt 22 overvægtige mænd med metabolisk syndrom, som under en højde forsøg blev flyttet fra 500 m til 1700 m over havets overflade i 3 uger. Gennem forsøget øgedes deres serum EPO, men ikke deres hæmoglobin, signifikant, og de tabte ca. 0,5 kg fedtmasse (Gunga et al 2000) og oplevede forbedringer i forskellige metaboliske parametre, herunder glukosefølsomhed og insulinresistens (Schobersberger et al 2000). Imidlertid er ændringer i fedtmasse efter Epo-behandling endnu ikke blevet undersøgt hos mennesker, og det er endvidere uvist, om sådanne metaboliske virkninger af Epo kan spille en rolle ved behandling af affektive lidelser.

Formål og hypoteser

Undersøgelse 1: Virkningen af Epo behandling på neurokognitive funktioner og depressive symptomer hos patienter med behandlingsresistent depression
At undersøge om en gentagen administration af Epo versus placebo til patienter med behandlingsresistent depression kan forbedre stemningslejet og genoprette de neurokognitive deficit målt ved neuropsykologisk testning, funktionel MR og kliniske humørskalær. Det er vores hypotese, at Epo vs. placebo-behandling vil have en antidepressiv virkning, forbedre kognitive funktioner og reducere processering af negative informationer.

Undersøgelse 2: Virkningen af Epo behandling på neurokognitive funktioner hos patienter med bipolar lidelse i remitteret fase
At undersøge, om ugentlig Epo-behandling i 8 uger har gavnlig virkning på neurokognitive funktioner hos patienter med bipolar lidelse i remitteret fase. Det er vores hypotese, at Epo vs. placebo-behandling vil forbedre kognitive funktioner og reducere processering af negative informationer.

Forsøgsplan og -design

Primære, sekundære og tertiære endpoints

Undersøgelse 1

Primært endpoint:	Antidepressiv effekt bedømt ved Hamilton Depression Scale 17 items (HAMD-17) (Hamilton 1960)
Sekundært endpoint:	Antal remissioner målt med HAMD-17
Tertiære endpoints:	Hukommelse og eksekutive funktioner, målt med henholdsvis Rey Auditory Verbal Learning Test (RAVLT) og Rapid Visual Information Processing (RVIP), perception af emotionelle ansigtsudtryk i en ansigtsgenkendelsestest, CPFQ samt depressive symptomer og livskvalitet vurderet ved Beck Depression Inventory (BDI) (Beck et al 1961), Positive And Negative Affect Scales (PANAS), WHO Quality of life BREF (WHOQOL-Bref) (WHO 1993), EuroQol visual analogue scale for livskvalitet (EQ-5D) (EuroQol 1990) og Global Assessment of Function (GAF) skala (Hall 1995).

Undersøgelse 2

Primært endpoint:	Hukommelse målt med RAVLT
Sekundære endpoints:	Vedvarende opmærksomhed og perception af negative ansigtsudtryk målt med hhv. RVIP og en ansigtsgenkendelsestest
Tertiære endpoints:	Stemningsleje, symptomer og livskvalitet målt med hhv. PANAS, HAMD-17, BDI, Young Mania Scale (YMS) (Young et al 1978), WHOQOL-Bref, EQ-5D, GAF og CPFQ.

Udforskning af mulige biomarkører:

1. Neurokognitive funktioner*.* Øget hippokampal aktivering efter en enkelt dosis af Epo (40,000 IU) til raske individer (Miskowiak et al 2007a), kan skyldes øgning af BDNF og neural plasticitet (Hariri et al 2003; Viviani et al 2005). Vi ønsker derfor at undersøge, om en mulig forbedring af hukommelsen efter gentagen Epo administration korrelerer med øget hippokampal aktivitet under billedgenkaldelse hos patienter med affektive lidelser. Endvidere ønsker vi at undersøge, om en eventuel antidepressiv virkning af Epo-behandling ledsages af reduceret kognitivt og neuralt respons på angste ansigtsudtryk ligesom nuværende antidepressiv behandling (Sheline et al 2001), hvilket kunne pege på modulering af disse bias som en central virkningsmekanisme. Struktuel MR-skanning vil endvidere blive foretaget for at undersøge, om Epo-behandling fører til strukturelle forandringer i hippokampus. MR-skanning foretages på en Siemens Trio skanner opererende på 3.0 T på MR-afdelingen, Hvidovre Hospital. Funktionelle MR data vil blive præ-processeret og analyseret med FEAT (FMRIB Expert Analysis Tool) version 5.43, som er en del af FSL (FMRIB Software Library version 3.3) (www.fmrib.ox.ac.uk/fsl).
2. Virkningen af Epo på perifer BDNF og inflammatoriske markører. Epo øger hippokampal BDNF og har anti-inflammatoriske virkninger (jf. punkt 2.2), men det er uvist, om dette er virkningsmekanismer for eventuel antidepressiv virkning af Epo. Vi ønsker derfor

med at undersøge effekten af Epo på plasma- og fuldblods-BDNF og inflammatoriske markører, IL-6 og CRP ved baseline (uge 1), 5, 9 og 14. På grund af en betydelig døgnvariation i perifær BDNF vil blodprøvetagning foregå på omtrent samme tidspunkt i døgnet hos alle patienter. Plasma og fuldblodsmåling af BDNF: blodprøver tages i EDTA-rør (Lilla) og centrifugeres umiddelbart. (Alternativt stilles i is-bad i max 60 min) Prøven centrifugeres i 15 min ved 4 grader C, ved 2500 g. Plasma pipetteres fra og stilles på frys (-20 °C), flyttes til −80 °C ved dagens afslutning. Plasma- og fuldblods-BDNF vil derefter blive analyseret ved brug af et kommersielt tilgængeligt to-sidet sandwich enzym-bundet immunabsorbent assay (ELISA) kit (Chemicon, Temecula, USA og IBL international, Hamburg).

3. Metabolisme. Præklinisk forskning peger på, at Epo reducerer fedtmasse og har en positiv virkning på metaboliske parametre (Hojman et al, submitted), hvilket kan spille en rolle ved behandling af affektive lidelser (McIntyre et al 2007). Vi ønsker derfor at undersøge virkningen af Epo på metaboliske parametre (faste glukose, faste insulin, HgbA1c, HDL-cholesterol, LDL-cholesterol og TAG) hos patienter med affektive lidelser ved hjælp af blodprøver ved baseline (uge 1), 5, 9 og 14 samt en DXA-skanning (Lunar Prodigy, GE Medical Systems Wisconsin, USA, version 8.8) ved baseline og uge 9. Med DXA-skanningen vurderes fedt- og fedtfri masse på hele kroppen, trunkus, arme og ben. Kvalitets-skanning bliver foretaget hver dag. Patienten skal ligge på et leje og en skannerarm kører langsomt hen over personen/patienten. Ved analyse vil ekstremiteter adskilles fra resten af kroppen ved hjælp af klare knogle fixpunkter. På denne måde vil vi være i stand til at vurdere Epos effekt på kropssammensætningen og fedtfordeling.

Metoder

Patienter

Undersøgelse 1: 40 patienter med behandlingsresistent depression i alderen 18-65 år, med et score på ≥ 17 på HAMD-17. Behandlingsresistens er defineret som manglende respons på antidepressiv medicin fra mindst to forskellige antidepressive klasser. Patienternes antidepressive behandling skal være uændret i to uger forud for undersøgelsen.

Antallet af patienter er estimeret på baggrund af en statistisk styrkeberegning ud fra HAMD scorer i vores tidligere studie af virkningen af en enkelt dosis af Epo (40.000 IU) vs. placebo hos depressive patienter (Miskowiak et al, under udarbejdelse). Den kliniske relevante forskel mellem behandlingsgrupperne på HAMD-17 (primært outcome) anses for at være mindst 3 point. For at opnå en styrke på 0.8 til påvisning af en sådan forskel mellem grupperne ved et alfa-niveau på $p>0.05$, skal der inkluderes $n=40$ patienter.

Undersøgelse 2: 40 patienter med bipolar lidelse i remitteret fase i alderen 18-65 år med et score på ≤ 14 på HAMD-17 og ≤ 14 på Young Mania Scale (YMS) (Young et al 1978), som har subjektive klager over kognitive problemer i moderat til svær grad indenfor mindst 2 ud af 7 mulige kognitive domæner på Massachusetts General Hospital Cognitive and Physical Functioning Questionnaire (Fava et al 2006) (sv.t. score ≥ 4 på disse domæner).

En statistisk styrkeberegning baseret på RAVLT scorer efter en enkelt dosis af Epo vs. placebo til depressive patienter (Miskowiak et al, under udarbejdelse) viser, at en stikprøvestørrelse på $n=40$ patienter vil have en styrke på > 0.8 til at påvise en forskel i RAVLT scorer (primært outcome) ved et alfa-niveau på $p<0.05$ efter gentagen Epo vs. placebo administration.

Rekruttering og screening
Patienter med behandlingsresistent depression (undersøgelse 1) og patienter med bipolar lidelse i remitteret fase, men med kognitive problemer, (undersøgelse 2) vil blive rekrutteret via Kompetencecenter for Affektive lidelser, Psykiatrisk Center, Rigshospitalet, Region Hovedstadens Psykiatri. Centeret modtager patienter med behandlingsresistent depression eller andre komplekse depressive tilstande fra hele Region Hovedstaden. Forsøgsdeltagere vil blive givet et informationshæfte og mulighed for at stille spørgsmål, før de tager stilling til deltagelse i undersøgelsen. De kan opnå yderligere information om undersøgelsen fra enhver af de navngivne forskere på projektet på hvilket som helst tidspunkt. Patienterne vil blive screenet med en medicinsk undersøgelse og psykiatrisk interview ved hjælp af Schedules for Clinical Assessment in Neuropsychiatry (SCAN; Wing et al 1990) for at bekræfte diagnosen og HAMD-17 / YMS for at bestemme sygdomsgraden.

Eksklusionskriterier er: skizofreni og stof- eller alkohol misbrug, rygning (> 5 cigaretter dagligt), betydelig selvmordsrisiko, diabetes, overvægt (BMI > 30), epilepsi eller epilepsi hos førstegradsfamilie, hæmatokritværdi > 50% for mænd og > 48% for kvinder, hypertension (blodtryk>160/100; gennemsnit af 3 målinger med 2 min interval), hjertekarsygdomme, tidligere tromboser eller tromboser hos førstegradsfamilie, tidligere eller aktuel kræftlidelse, graviditet, p-piller eller tidligere Epo-behandling. Der gives forbud mod indtagelse af jern-tilskud under studiet, da dette ville føre til uhensigtsmæssig stigning i hæmatokrit og risiko for tromboser og hypertension. Der tillades antidepressiva, lithium eller antipsykotisk medicin. En graviditetstest tages for kvindelige deltagere i deres reproduktive år. Blodscreening foretages (hæmoglobin, hæmatokrit, erytrocytter, retikulocytter, trombocytter, MCV, MCH, bilirubin, alkalisk fosfatase, ALAT, kreatinin) før studiet, hver uge under Epo behandling og herefter ved uge 9, 10 og 14 for at overvåge patienternes tilstand og kontrollere for bivirkninger ved Epo-behandlingen. Alle deltagere vil blive bedt om at give skriftligt, informeret samtykke og blive forklaret, at de kan afbryde deres deltagelse i undersøgelsen på ethvert tidspunkt uden at give nogen forklaring, og uden at det vil påvirke deres fortsatte behandling i centeret. Information om deltagelse i undersøgelsen vil blive tilsendt patienternes praktiserende læge for at undgå potentielle medicinske komplikationer.

Randomisering
Stratificering og randomisering af patienterne er foretaget af Pharma Consulting Group (http://www.pharmaconsultinggroup.com/) med stratificering for køn og alder (over eller under 35 år).

Forsøgsdesign
Forsøgsdeltagerne vil blive randomiseret til at modtage enten Epo eller saltvand intravenøst, som tillæg til vanlig behandling, og i et dobbelt-blindt imellem-gruppe design ugentligt i 8 uger. Neuropsykologisk testning gennemføres ved baseline og uge 9 og 14 (se detaljer nedenfor). Stemningsleje og symptomer vil blive monitoreret ved baseline og uge 5, 9 og 14 (se nedenfor). MR-skanning vil blive foretaget ved baseline og uge 14 (se oversigtstabel nedenfor).

Neuropsykologiske tests og kliniske ratingskalaer
Kognitive funktioner vil blive undersøgt med et neuropsykologisk testbatteri, der måler styringsfunktioner, opmærksomhed og psykomotorisk tempo, hukommelse og emotionel processering. Batteriet indeholder følgende tests: Rey Auditory Verbal Learning Test (RAVLT), Trail making test A and B, RBANS digit span, RBANS coding, WAIS-III letter number sequencing, ordmobilisering, ansigtsgenkendelse og samt følgende computeriserede CANTAB tests (Cambridge Cognition Ltd.): Simple Reaction Time, Rapid Visual Processing, Delayed Match to Sample og Spatial Working Memory. I forbindelse med den funktionelle MR-skanning (baseline og uge 14) vil det neurale grundlag for arbejdshukommelse, hukommelse og emotionel processering blive undersøgt med hhv. (1) en N-back arbejdshukommelsesopgave, (2) en billedindkodning – genkaldelsesopgave samt (3) en ansigtsprocesseringstest.

Stemningsleje, symptomer og livskvalitet bliver vurderet med HAMD-17, Symptomvurderingsskemaet baseret på Beck Depression Inventory (BDI) (Beck et al 1961), Hospital Anxiety and Depression Scale (HADS) (Zigmond & Snaith 1983), Young Mania Scale (YMS) (Young et al 1978), WHO Quality of life BREF (WHOQOL-Bref) (WHO 1993), Positive And Negative Affect Scales (PANAS) (Watson et al 1988) og EuroQol visual analogue scale (VAS) for livskvalitet (EQ-5D) (EuroQol 1990). Måling af serum – og plasma BDNF foretages ved baseline og uge 5, 9 og 14. På grund af en betydelig døgnvariation i serum og plasma BDNF vil tidspunkt for blodprøvetagning blive registreret, og det tilsigtes at disse, så vidt muligt, foregår i et bestemt tidsrum på døgnet (formiddag) for alle patienter.

Oversigtstabel
Tidspunkter for måling og registrering af primære, sekundære og tertiære endpoints fremgår af nedenstående oversigtstabel. Effektparametre analyseres efter undersøgelsernes afslutning.

	**Interventions / Assessment domains**	**Tests/Evaluations**	**Screening**	**Baseline & treatment start***	**Active treatment phase**	**Treatment-free follow-up period**
				Week 1	(Week 2-8)	(Week 9-14)
**Treatment**	Investigational drug	EPO (40,000 IU)/ Placebo	-	1x	7x (weekly)	-
**Efficacy**	Neurocognitive functions	Neuropsychological testing (120 min)	-	1x	-	2x (week 9,14)
		MRI, fMRI and cognitive tests	1x	-	-	1x (week 14)
	Psychopathology	Clinical Rating	-	1x	1x (week 5)	2x (week 9,14)
		Questionnaires	-	1x	1x (week 5)	2x (week 9,14)
	Quality of life questionnaires		-	1x	1x (week 5)	2x (week 9,14)
	DXA scan		-	1x	-	1x (week 9)
**Safety**	Physical exam		1x	1x	7x (weekly)	3x (week 9,10,14)
	Vital signs		1x	1x	7x (weekly)	3x (week 9,10,14)
	Urine pregnancy test		1x	1x	3x (week 3,5,7)	-
	ECG		1x	-	2x (week 3,5)	-
	Adverse event monitoring		-	*Continuously until week 10*		
	Blood analysis	Haematology	1x	1x	7x (weekly)	3x (week 9,10,14)
		Blood chemistry	1x	1x	7x (weekly)	3x (week 9,10,14)
		BDNF, inflammatory and metabolic markers	-	1x	1x (week 5)	2x (week 9,14)

** All tests to take place prior to drug administration during the same visit at the clinic (drug treatment is on the first day of week)*

Udtagelse og opbevaring af biologisk materiale

Blodprøver fra undersøgelsen vil indgå i Dansk Psykiatrisk Biobank, godkendt af Videnskabsetisk Komite (journalnr. KF 01-024/01).

Bivirkninger, risici, ulemper og tilhørende sikkerhedsforanstaltninger

Epo er vidt brugt og har en udmærket sikkerhedsmæssig profil i behandling af anæmi, hvor det oftest gives i lav-dosis over en periode af uger eller måneder for at øge antallet af røde blodlegemer. Epo i høj-dosis på 40.000 IU (Miskowiak et al 2007a, 2007b, 2008a, 2008b) og på 15.000 IU (Lundby et al 2008) givet til henholdsvis 24 og 11 raske mennesker er fundet veltolereret og uden bivirkninger. Gentagen høj-dosis administration (33.000 IU) til 600 patienter med iskæmisk hjerneinfarkt (jf. Ehrenreichs vedlagte samarbejdsbrev; Ehrenreich et al 2002), ugentlig høj-dosis Epo-administration (40.000 og 48.000 IU) over flere måneder til 60 patienter med skizofreni og multipel sklerose (jf. Ehrenreichs vedlagte samarbejdsbrev; Ehrenreich et al 2007a, 2007b), og langtidsbehandling (14 uger) med Epo (5.000 IU) af 8 raske frivillige (Lundby et al 2008) er ligeledes fundet veltolereret.

Nogle potentielle akutte bivirkninger af Epo-administration er forbigående influenza-lignende symptomer, udslæt ved injektionsstedet og hovedpine, som normalt rapporteres som mild og forsvinder inden for få timer. Forsøgsdeltagerne forbliver derfor under lægelig observation i mindst 1 time efter Epo/saltvands-indsprøjtningen på Psykiatrisk Center, Rigshospitalet.

Langtidsbehandling med Epo kan føre til forhøjet blodtryk og til øget risiko for blodpropper og epileptiske anfald i medicinsk dårlige patienter med kronisk nyresvigt. I det nærværende studium udelukkes derfor individer, som selv eller hvis førstegradsfamilie har en historie af tromboser eller epilepsi, og patienter med forhøjet blodtryk, diabetes, hjertekarsygdomme eller som tager p-piller. Derudover bliver patienter monitoreret med ugentlige medicinske undersøgelser, blodtryksmålinger og blodprøver under studieforløbet og indtil 2 uger efter dets afslutning for at sikre, at patienternes generelle helbred og blodparametre (trombocyttal) forbliver normale. Skulle der forekomme blodtrykstigning i forbindelse med Epo-behandling, vil patienterne modtage rette medicinske behandling og/ eller blive udelukket fra yderligere behandling (jf. procedurerne beskrevet i Ehrenreichs vedlagte samarbejdsbrev). I tilfælde af betydelig stigning i trombocyttal, vil Epo-behandlingen blive afbrudt og patienten fulgt op med ugentlige blodprøver og medicinske undersøgelser, indtil trombocytniveauet er normaliseret (ibid.).

Der er ikke observeret hæmatokrit-stigning ved akut Epo administration (40.000 IU/ 15.000 IU) til 35 frivillige (Miskowiak et al 2007, 2008; Lundby et al 2008) eller ved 3 doser Epo (33.000 IU) over 3 efterfølgende dage til 33 patienter med iskæmisk hjerneinfarkt (Ehrenreich et al 2002). Derimod observeredes der hæmatokritstigning hos 25 ud af 25 patienter efter 5 ugers ugentlig Epo-behandling (40.000/ 48.000 IU) (Ehrenreich et al 2007a, 2007b). Der var hos 11 af disse patienter brug for enkelte åreladninger mellem uge 5-8 ved en hæmatokritværdi på >50% for mænd og >48 % for kvinder ved 2 ugentlige på hinanden efterfølgende målinger; 7 patienter 1 gang, 1 patient 2 gange, 2 patienter 3 gange og 1 patient 5 gange (ibid.). Åreladning er en mindre, ganske ufarlig procedure (som en bloddonation), der i studierne var uden betydeligt ubehag for patienterne (Ehrenreich, personlig kommunikation; jf. Ehrenreichs vedlagte samarbejdsbrev). Fortsat ugentlig Epo-behandling af disse patienter var veltolereret og førte efter uge 8 ikke til yderligere hæmatokritstigning. Dette var forudsat, at patienterne ikke indtog jern-tilskud under Epo-behandlingen (jf. Ehrenreichs samarbejdsbrev). I det aktuelle studie gives der derfor ligeledes forbud mod indtagelse af jern-tilskud. Vi forventer, at der vil være behov for åreladning mellem uge 5-8 hos omkring 40% af Epo-behandlede patienter (8 patienter per studie), hvoraf de fleste kun vil behøve en enkelt åreladning (Ehrenreich personlig kommunikation). Disse patienter vil, uden afbrydelse af behandlingen (jf. Ehrenreich et al 2007a, 2007b; Ehrenreichs vedlagte samarbejdsbrev), blive fulgt op, indtil hæmatokrit-niveauet er normaliseret. Vi har etableret et tæt samarbejde med professor Ehrenreich (jf. vedlagte samarbejdsbrev), som har erfaring med langtidsbehandling med Epo af omkring 60 patienter. Dermed kan vi sikre os den samme sikkerhedsprocedure og tætte monitorering af patienternes blodtal med ugentlige blodprøver og eventuelle åreladninger.

En meget sjælden, men alvorlig bivirkning af Epo behandling er en tilstand, hvor patienter udvikler en autoimmun reaktion og deraf følgende "pure red cell aplasia" (PRCA). Til dags dato er Epo-induceret PRCA næsten udelukkende associeret med subkutan (under huden) administration. Dette problem er stort set løst gennem ændringer i Epo-pakning og

præparation introduceret af producenterne samt mere nøjsommelig overholdelse af nedkølingsprocedurer ved Epo-transport og -opbevaring (jf. Ehrenreich's samarbejdsbrev). I lyset af dette og eftersom det aktuelle studie ikke involverer subkutan administration, anses risikoen for PRCA for at være forsvindende lille. Ved blodscreeningen vil Epo-antistof niveauet dog blive målt som ekstra sikkerhedsprocedure (jf. Ehrenreich's samarbejdsbrev). Patienter vil blive informeret om alle risici ved Epo administration, før de giver deres informerede samtykke. Til trods for den forsvindende lille risiko for PRCA bliver forsøgspersonerne opfordret til straks at kontakte en læge, der er tilknyttet til studiet m.h.p. diagnostik og evt. behandling, hvis de skulle opleve uvant søvnighed, tab af energi og bleghed. Hvis Epo-antistoffet måles, bliver Epo-behandlingen afbrudt og den rette medicinske behandling igangsat.

Der findes ingen publicerede undersøgelser af Epos virkning hos patienter med bipolar lidelse (hverken i akut eller remitteret fase). Der er dog ud fra vores tidligere undersøgelse (Miskowiak et al, under udarbejdelse) intet, der tyder på, at patienter i den depressive fase af bipolar lidelse skulle udvikle mani eller opleve andre uhensigtsmæssige virkninger af Epo.

Videnskabsetisk redegørelse

Fordele

Nuværende behandlingsstrategier for depression og bipolar lidelse har stadig mangler, og der er ifølge WHO et presserende behov for udvikling af nye, mere effektive behandlingstilbud. Meget tyder på, at medicin, der har en hurtigt indsættende virkning på neuroplasticitet kan repræsentere en ny generation af antidepressiva. Epo besidder sådanne neuroregenerative egenskaber, og vi har vi påvist gavnlig effekt af Epo på neurokognitive funktioner og humøret hos raske mennesker. Det er dog hidtil ikke undersøgt, om Epo-behandling har antidepressiv virkning og forbedrer neurokognitive funktioner hos patienter med affektive lidelser. De aktuelle undersøgelser vil derfor ikke blot bidrage til vores videnskabelige forståelse af, hvordan Epo påvirker den menneskelige hjerne, og hvilken rolle neuroplasticitet spiller i forbindelse med depression og mani. Studierne vil også kunne medvirke til udvikling af nye, bedre behandlingstilbud med betydning for patienternes livskvalitet og arbejdsdygtighed i fremtiden.

Forsøgsdeltagerne i de aktuelle studier er patienter med behandlingsresistent depression (studie 1) og patienter med tidligere maniske og/eller depressive episoder, som har vedvarende problemer med hukommelse og planlægning (studie 2). For begge patientgrupper er der fordele at deltage i undersøgelsen. Ved behandlingsresistent depression (defineret som manglende effekt af mindst 2 typer antidepressiv medicin) er der ikke videnskabelig evidens for effekt af en tredje farmakologisk behandling. Disse patienter vil derfor kunne få gavn af nye potentielle antidepressiva som Epo, der har en direkte, hurtigt indsættende effekt på hjernens evne til at regenerere og danne nye hjerneceller. For patienter med tidligere mani og/eller depression vil Epo-behandlingen kunne reducere hukommelses og planlægningsproblemer til gavn for patienternes arbejdsdygtighed og livskvalitet. I begge studier har patienterne 50% chance for at få den aktive Epo-behandling og eventuel forbedring af stemningslejet og kognitive funktioner. Endvidere vil alle patienter gennem deres deltagelse i undersøgelsen få gavn af tæt læge- og psykolog-kontakt under hele studieforløbet. Hvis undersøgelsen viser positiv virkning af Epo på stemningslejet og kognitive funktioner, vil Epo være kunne blive et reelt behandlingstilbud til disse patientgrupper i fremtiden.

Ulemper

Som beskrevet i afsnittet "Bivirkninger, risici, ulemper og tilhørende sikkerhedsforanstaltninger" er der nogle risici og ulemper ved deltagelse i undersøgelsen. Vi mener imidlertid, at disse er minimale set i lyset af sikkerhedsdata fra tidligere Epo undersøgelser og de beskrevne forholdsregler, eksklusionskriterier og nøje opstillede behandlingsplan i tilfælde af bivirkninger. Ehrenreich skriver i sit samarbejdsbrev (vedlagt): "I clearly encourage you to go ahead and perform this study since EPO may have a lasting beneficial effect on affective and cognitive symptoms in this patient population and the risk is

minimal, provided the above delineated careful risk management is guaranteed." I overensstemmelse med Epos gode sikkerhedsprofil har Videnskabsetisk Komité for København og Frederiksberg Kommuner tidligere godkendt undersøgelser af akut og langtids- Epo-behandling (14 uger) af raske frivillige (KF 01-269-637 og KF-01-291917).

Skønt patienterne i de aktuelle studier ikke vil blive frataget deres vanlige antidepressive eller stemningsstabiliserende behandling, kræver studiet, at patienterne ikke ændrer behandling under studieforløbet. Dette kan give anledning til etiske overvejelser for de depressive patienter i undersøgelse 1. Imidlertid er disse patienter karakteriseret af behandlingsresistens for nuværende antidepressiv medicin, og det er uafklaret, hvilken behandling, der bør kan hjælpe denne patientgruppe. Vi mener derfor, at det er helt etisk forsvarligt ikke at foretage ændringer i patienternes vanlige antidepressive medicin under de 8 ugers Epo/ placebo behandling.

Økonomi og vederlag

Forsøgspersoner modtager vederlag for tabt arbejdsfortjeneste enten dokumenteret eller svarende til faglært arbejder samt transportgodtgørelse i forbindelse med deltagelse i undersøgelsen.

Øvrig anmeldelse af undersøgelsen

Projektet er godkendt af Datatilsynet (2008-41-2711) og af Lægemiddelstyrelsen .

Monitorering af undersøgelsen

GCP-enheden ved Københavns Universitetshospital vil monitorere undersøgelsen for at sikre at den lever op til forskrifterne for Good Clinical Practice.

Økonomiske forhold

Psykolog, ph.d. Kamilla Miskowiak har skaffet økonomisk støtte til undersøgelsen fra Forskningsrådet for Sundhed og Sygdom (FSS) (1,9 mio. DKK) og Novo Nordisk Fonden (350.000 DKK). Der er indsendt ansøgning til Lundbeckfonden til dækning af omkostninger i forbindelse med funktionel MR-skanning (svar forventes i september 2009).

Offentliggørelse af resultater

Såvel negative som positive resultater af undersøgelserne vil blive publiceret. Publikationerne vil generelt have følgende forfatterrækkefølge: førsteforfatter: Kamilla Miskowiak, andenforfatter: Maj Vinberg, sidsteforfatter: Lars Kessing; dog med undtagelse af publikationen om virkningen af Epo på serum/plasma BDNF, hvor Maj Vinberg er førsteforfatter og Kamilla Miskowiak andenforfatter (Lars Kessing sidsteforfatter). Resten af medforfatterne vil blive fordelt herimellem i forhold til deres bidrag til projektet og udfærdigelsen af publikationerne.

Retningslinier for indhentelse af informeret samtykke

Patienter vil blive rekrutteret enten ved udsendelse af skriftlig information om undersøgelsen og invitation til at deltage (se bilag) eller ved en samtale med de navngivne forskere på disses kontor i forbindelse med patienternes besøg på Kompetencecenter for Affektive lidelser, Psykiatrisk Center, Rigshospitalet, Region Hovedstadens Psykiatri. Forsøgsdeltagere vil blive givet et informationshæfte og mulighed for at stille spørgsmål, før de tager stilling til

deltagelse i undersøgelsen. De kan opnå yderligere information om undersøgelsen fra enhver af de navngivne forskere på projektet på hvilket som helst tidspunkt. Patienterne bliver tilbudt at have en bisidder ved informationssamtalen. Der rekrutteres kun ambulante patienter, som er myndige, habile og i stand til at forstå de givne mundtlige og skriftlige informationer. Alle patienter vil inden deres deltagelse blive bedt om at give skriftligt, informeret samtykke og blive forklaret, at de kan afbryde deres deltagelse i undersøgelsen på ethvert tidspunkt uden at give nogen forklaring og uden at det vil påvirke deres fremtidige behandling i centeret. De vil blive givet mindst 24 timers betænkningstid mellem den mundtlige og skriftlige information og senere underskrift på samtykke– og fuldmagtserklæringen.

Tidsplan for forskningen
Aug 2009 – Sep 2011: Rekruttering og testning af patienter med behandlingsresistent depression (undersøgelse 1)
Sep 2011 – Sep 2013: Rekruttering og testning af patienter med bipolar lidelse i remitteret fase (undersøgelse 2) *
Sep 2011 – Sep 2014: Analyse, dissemination, publikation af artikler og præsentation af resultater ved internationale konferencer.

**Af praktiske rekrutteringshensyn vil der være et vist tidsmæssigt overlap mellem de to studier*.

Generelle implikationer
De nærværende studier udforsker virkningen af Epo på neurokognitive funktioner og stemningsleje hos patienter med behandlingsresistent depression eller bipolar lidelse i remitteret fase. Hvis denne forskning påviser en gavnlig virkning af Epo på neuropsykologiske funktioner hos disse patientgrupper og forbedring af stemningslejet hos patienter med behandlingsresistent depression, vil det fremhæve Epo som kandidat til fremtidig "kognitiv enhancement" som tillægsbehandling ved depression og bipolar lidelse. Studierne vil således kunne få stor betydning for patienternes livskvalitet og deres sociale og arbejdsmæssige funktionsniveau.

Referencer

Agnello et al (2002): Erythropoietin exerts an anti-inflammatory effect on the CNS in a model of experimental autoimmune encephalomyelitis. Brain Res 952(1):128-34.
Austin et al (1992): Cognitive function in major depression. J Affect Disord 25:21-9
Belmaker and Agam (2008): Mechanisms of Disease: Major Depressive Disorder. N Engl J Med 358(1):55-68
Beck et al (1961): An inventory for measuring depression Arch Gen Psychiatry 4:561-71
Zigmond & Snaith (1983). The Hospital Anxiety And Depression Scale. Acta Psychiatr Scand 67:361-70
Berton and Nestler (2006): New approaches to antidepressant drug discovery: beyond monoamines. Nat Rev Neurosci 7:137-51
Bhagwagar et al (2004): Normalization of enhanced fear recognition by acute SSRI treatment in subjects with a previous history of depression. *Am J Psychiatry* 161(1):166-8
Brines and Cerami (2005): Emerging biological roles for erythropoietin in the nervous system. Nat Neurosci 6:484-94
Brines et al (2000): Erythropoietin crosses the blood-brain barrier to protect against experimental brain injury.Proc Natl Acad Sci U S A 97(19):10526-31
Browning et al (2004): A single dose of citalopram increases fear recognition in healthy subjects. J Psychopharmacol 21(7):684-90
Chamberlain and Sahakian (2006): The neuropsychology of mood disorders. Curr Psychiatry Rep 8:458-63
Chamberlain and Sahakian (2004): Cognition in mania and depression: psychological models and clinical implications. Curr Psychiatry Rep 6(6):451-8
Clark et al (1999): *Scientific Foundations of Cognitive Theory and Therapy of Depression*. John Wiley, New York
Clark et al (2005): Impairment of executive function but not memory in first-degree relatives of patients with bipolar I disorder and in euthymic patients with unipolar depression. Am J Psychiatry 162:1980-82
Clark and Goodwin (2004): State- and trait-related deficits in sustained attention in bipolar disorder. Eur Arch Psychiatry Clin Neurosci. 254(2):61-8
Cohen RM, Weingartner H, Smallberg SA, Pickar D, Murphy DL (1982): Effort and cognition in depression. Arch Gen Psychiatry 39:593-7
Cunha et al (2006): Serum brain-derived neurotrophic factor is decreased in bipolar disorder during depressive and manic episodes. Neurosci Lett 398(3):215-9
Dickerson et al (2001): Outpatients with schizophrenia and bipolar I disorder: Do they differ in their cognitive and social functioning? Psychiatry Res 102:21–27
Dickerson et al (2004): Cognitive functioning in schizophrenia and bipolar disorder: comparison of performance on the Repeatable Battery for the Assessment of Neuropsychological Status. Psych Res 129:45-53
Dion et al (1988): Symptoms and functioning of patients with bipolar disorder six months after hospitalization. Hosp Community Psychiatry 39: 652–657
Ehrenreich et al (2002): Erythropoietin therapy for acute stroke is both safe and beneficial. Mol Med 8:495-505
Ehrenreich et al (2007a): Improvement of cognitive function in chronic schizophrenic patients by recombinant human erythropoietin. Mol Psychiatry 12(2):206-20
Ehrenreich et al (2007b): Exploring recombinant human erythropoietin in chronic progressive multiple sclerosis. Brain 130(10):2577-88
Ekman and Friesen (1976): Pictures of facial affect. Palo Alto, Calif: Consulting Psychologists Press
Erbayraktar et al (2003): Asialoerythropoietin is a nonerythropoietic cytokine with broad neuroprotective activity in vivo. Proc Natl Acad Sci U S A 100:6741-6
EuroQol - a new facility for the measurement of health-related quality of life. The EuroQol Group. Health Policy 1990; 16(3):199-208

Fava et al (2006): A cross-sectional study of the prevalence of cognitive and physical symptoms during long-term antidepressant treatment. J Clin.Psychiatry 67: 1754-9

Fennig et al (2002): Everyday memory and laboratory memory tests: general function predictors in schizophrenia and remitted depression. J Nerv Ment Dis 190:677-82

Ferrario et al (2004): Treatment of cancer-related anemia with epoetin alfa: a review. Cancer Treat Rev 30:563-575

Frasch et al (2000): [Do cognitive deficits in depressive disorders remit?]. Psychiatr Prax 27:291-5

Frey et al (2007): The role of hippocampus in the pathophysiology of bipolar disorder. Behav Pharmacol.18:419-30

Gonul et al (2005): Effect of treatment on serum brain-derived neurotrophic factor levels in depressed patients. Eur Arch Psychiatry Clin Neurosci 255(6):381-6.

Grassi-Oliveira et al (2008): Low Plasma Brain-Derived Neurotrophic Factor and Childhood Physical Neglect Are Associated with Verbal Memory Impairment in Major Depression-A Preliminary Report. Biol Psychiatry (in press)

Hamilton (1960): A rating scale for depression. J Neurol Neurosurg Psychiatry 23:56-62

Hammar A, Lund A, Hugdahl K (2003): Long-lasting cognitive impairment in unipolar major depression: a 6-month follow-up study. Psychiatry Res 118: 189-96

Hariri et al (2003): Brain-derived neurotrophic factor val66met polymorphism affects human memory-related hippocampal activity and predicts memory performance. J Neurosci 23:6690-4

Harmer et al (2004): Increased positive versus negative affective perception and memory in healthy volunteers following selective serotonin and norepinephrine reuptake inhibition. Am J Psychiatry 161:1256-63

Harmer et al (2006): Antidepressant Drug Treatment Modifies the Neural processing of Nonconscious Threat Cues. Biol Psychiatry 59:816-20

Karege et al (2002a): Postnatal developmental profile of brain-derived neurotrophic factor in rat brain and platelets. Neurosci Lett 328:261–4

Karege et al (2002b): Decreased serum brain-derived neurotrophic factor levels in major depressed patients. Psychiatry Res 109(2):143-8.

Karege et al (2005): Low brain-derived neurotrophic factor (BDNF) levels in serum of depressed patients probably results from lowered platelet BDNF release unrelated to platelet reactivity. Biol Psychiatry 57(9):1068-72

Kessing (1998): Cognitive impairment in the euthymic phase of affective disorder. Psychol Med 28:1027-38

Kessing & Andersen (2004): Does the risk of developing dementia increase with the number of episodes in patients with depressive disorder and in patients with bipolar disorder? *J Neurol Neurosurg Psychiatry* 75:1662-6

Lee et al (2006): Erythropoietin reduces perihematomal inflammation and cell death with eNOS and STAT3 activations in experimental intracerebral hemorrhage. J Neurochem 96(6):1728-39

Leist et al (2004): Derivatives of erythropoietin that are tissue protective but not erythropoietic. Science 305:239-42

Lundby et al 2008: Erythropoietin receptor in human skeletal muscle and the effects of acute and long-term injection with recombinant human erythropoietin on the skeletal muscle. J Allp Physiol 104:1154-60

Machado-Vieira et al (2008): Rapid Onset of Antidepressant Action: A New Paradigm in the Research and Treatment of Major Depressive Disorder. J Clin Psychiatry (in press)

Manji & Duman (2001): Impairments of neuroplasticity and cellular resilience in severe mood disorders: implications for the development of novel therapeutics. Psychopharmacol Bull 35(2):5-49.

Marcos et al (1994): Cognitive dysfunctions in recovered melancholic patients. *J Affect Disord* 32:133-7

Martínez-Arán et al (2000): Cognitive dysfunctions in bipolar disorder: evidence of neuropsychological disturbances. Psychother Psychosom 69(1):2-18.

Marvel & Paradiso (2004): Cognitive and neurological impairment in mood disorders. Psychiatr Clin North Am. 27:19-36
Miskowiak et al (2007a): Erythropoietin enhances hippocampal response during memory retrieval in humans. J Neurosci 27(11):2788-92
Miskowiak et al (2007b): Erythropoietin reduces neural and cognitive processing of fear in human models of antidepressant drug action. Biol Psychiatry 62(11):1244-50
Miskowiak et al (2008a): Erythropoietin improves mood and modulates the cognitive and neural processing of emotion three days post administration. Neuropsychopharmacology 33(3):611-8
Miskowiak et al (2008b): Differential effects of erythropoietin on neural and cognitive measures of executive function 3 and 7 days post-administration. Exp Brain Res 184(3):313-21
Mitoma et al (2008): Stress at work alters serum brain-derived neurotrophic factor (BDNF) levels and plasma 3-methoxy-4-hydroxyphenylglycol (MHPG) levels in healthy volunteers: BDNF and MHPG as possible biological markers of mental stress? Prog Neuropsychopharmacol Biol Psychiatry 32(3):679-85.
Moffoot AP, O'Carroll RE, Bennie J, Carroll S, Dick H, Ebmeier KP, et al (1994): Diurnal variation of mood and neuropsychological function in major depression with melancholia. J Affect Disord 32:257-69
Morishita et al (1997): Erythropoietin receptor is expressed in rat hippocampal and cerebral cortical neurons, and erythropoietin prevents in vitro glutamate-induced neuronal death. Neuroscience 76:105–16
Neu et al (2005): Cognitive function over the treatment course of depression in middle-aged patients: correlation with brain MRI signal hyperintensities. J Psychiatr Res 39:129-35
Paradiso et al (1997): Cognitive impairment in the euthymic phase of chronic unipolar depression. J Nerv Ment Dis:185:748-54
Ransome and Turnley (2007): Systemically delivered Erythropoietin transiently enhances adult hippocampal neurogenesis. J Neurochem 102(6):1953-65
Rauch et al (2003): Neuroimaging studies of amygdala function in anxiety disorders. *Ann N Y Acad Sci* 985:389-410
Rosenzweig-Lipson et al (2007): Differentiating antidepressants of the future: efficacy and safety.Pharmacol Ther 113(1):134-53
Sakanaka et al (1998): In vivo evidence that erythropoietin protects neurons from ischemic damage. Proc Natl Acad Sci USA 95:4635–40
Santarelli et al (2003): Requirement of hippocampal neurogenesis for the behavioral effects of antidepressants. Science 301:805-9
Sättler et al (2004): Neuroprotective effects and intracellular signaling pathways of erythropoietin in a rat model of multiple sclerosis. Cell Death Differ 11(2):S181-92
Shimizu et al (2003): Alterations of serum levels of brain-derived neurotrophic factor (BDNF) in depressed patients with or without antidepressants. Biol Psychiatry 54(1):70-5
Shirayama et al (2002): Brain-derived neurotrophic factor produces antidepressant effects in behavioral models of depression. J Neurosci. 22:3251-61
Spinhoven et al (2006) Autobiographical memory in the euthymic phase of recurrent depression. J Abnorm Psychol 115:590-600
Tavares JV et al (2003): Cognition in mania and depression. Psychol Med 33:959-67
Taylor Tavares et al (2007): Distinct profiles of neurocognitive function in unmedicated unipolar depression and bipolar II depression. Biol Psychiatry 62(8):917-24
Trichard et al (1995) Time course of prefrontal lobe dysfunction in severely depressed in-patients: a longitudinal neuropsychological study. Psychol Med 25:79-85
Viviani et al (2005): Erythropoietin protects primary hippocampal neurons increasing the expression of brain-derived neurotrophic factor. J Neurochem 93:412-42
Wang et al (2004): Treatment of stroke with erythropoietin enhances neurogenesis and angiogenesis and improves neurological function in rats. Stroke 35:1732-7
Watson et al (1988): Development and validation of brief measures of positive and negative affect: The PANAS scales. J Pers Soc Psychol 54:1063-70

Wing et al (1990): SCAN. Schedules for Clinical Assessment in Neuropsychiatry. Arch Gen Psychiatry 47:589-93

Yatsiv et al (2005): Erythropoietin is neuroprotective, improves functional recovery, and reduces neuronal apoptosis and inflammation in a rodent model of experimental closed head injury. FASEB J. 19(12):1701-3

Young et al (1978): A rating scale for mania: reliability, validity and sensitivity. Br J Psychiatry133:429-35

Yukimasa et al (2006): High-frequency repetitive transcranial stimulation improves refractory depression by influencing catecholamine and brain-derived neurotrophic factors. Pharmacopsychitry 2006;39:52–9

Yoshimura et al (2007): Effects of paroxetine ormilnacipran on serumbrain-derived neurotrophic factor in depressed patients. Prog Neuropsychopharmacol Biol Psychiatry 31:1034–7

Wing et al (1990): SCAN. Schedules for Clinical Assessment in Neuropsychiatry. Arch Gen Psychiatry 47:589-93

Weiland-Fiedler et al (2004): Evidence for continuing neuropsychological impairments in depression. J Affect Disord 82:253-8

WHOQOL Group, 1994. The development of the World Health Organisation quality of life assesment instrument (theWHOQOL). Springer, Berlin Heidelberg New York.

Zigmond & Snaith (1983). The Hospital Anxiety And Depression Scale. Acta Psychiatr Scand 67:361-70
